# Graduates of Rush Med. College, 1873–4

**Published:** 1874-05

**Authors:** 


### *Rush Medical College—Names of Graduates, 1873-4.*

NOTE.—The subjoined List of Graduates should have been published in the March No. of the JOURNAL, but the manuscript having been lost, it was unavoidably omitted until a correct copy could be obtained. The Editors trust that this accident will be deemed a sufficient apology to the Class for the apparent neglect.

William Andrew Allen.
Sanford Fillmore Bennett.
Victor Arthur Bertram.
Charles LeRoy Burroughs.
John Henry Byrne.
Oscar Nathan Carr.
Theodore Jefferson Catlin.
George Henry Chapman.
Frank Wilbur Chase.
Ira Broadwell Connett.
James Wells Cook.
James Edwin Cowan.
Henry Crowder.
Frederich Wilhelm Denke.
Robert Ford Dundass.
Leonidas Hamlin Eaton.
David William Edgar.
Andrew Judson Ervey.
William Henry Franks.
William Harrison French.
Ira Hamilton Gillum.
Ezra T. Goble.
Zenas Harmon Going.
George Washington Greaves.
William Samuel Grimes.
John Edgar Hathorn.
Truman Augustus Herrington.
Wilbur Alson Hendryx.
Gershom Hyde Hill.
Lewis Cass Hormell.
John Wesley Lane.
Abraham Leigh.
William Russel Lewis.
Robert A. Livingston.
Frank Howard Lord.
Henry Smith Lytle.
Herbert Marcus McKenzie.
Robert Edward McClelland.
Addison Webster McCoy.
James Harold McCune.
James Gallagher McElroy.
Oliver Harrison Martin.
Samuel Warren Mercer.
George Henry Miller.
Frank Lawrence Miles.
Theophilus Wells Mitchell.
Ellis Crosby Moore.
William Harrison Morgan.
Lea Murphy.
Ralph Parkin.
George Weston Parsons.
William Parsons.
Frank Howard Payne.
Weston Theodore Plumb.
Kossuth Fillmore Purdy.
Frank Allen Reed.
Addison Winfield Rickey.
Laurel Elmer Robison.
William Rofe.
Franklin LaFayette Rownd.
Joseph Augustus Scroggs.
Edgar Barber Shumway.
Archie Robertson Small.
Arthur Henry Steen.
Daniel Morrison Benonia Thom.
Edson Reuben Wait.
Lewis Franklin Walker.
Spencer Cone Wernham.
James Delaforet Whitley.
Constantine Wiley.
Thomas Royston Wiley.
Arthur Lee Wright.
William Henry Youngham.

*Honorary Degree.*— Dr. Rogers, Bloomington.

Dr. Willcox, Dr. Theodore J. Bluthardt, Chicago.

**Directory for the Month.**

*Every Monday. Lectures.*—At Rush College, 9 to 1 o'clock.—Drs. Wadsworth, Bridge, Jackson and Parkes. At the Chicago College, 8 to 12 o'clock. Drs. Quine, Curtis and Roler. At Woman's Hospital College, 9 to 11 o'clock. Drs. Dyas and Fitch.

*Clinics.*—At Mercy Hospital, 3 P. M. Surgery.—Dr. Andrews. At the Central Dispensary (239 W. Van Buren street), 3 P. M. Medical.—Dr. Bridge. At the Eye and Ear Infirmary, 2 P. M.—Dr. Holmes. At Hospital for Women and Children, 1.30 P. M. Gynæcological.—Dr. Thompson.

*Mondays, May 4th and 18th.*—Meeting of *Chicago Medical Society*, in the evening, at Gault House.

*Mondays, May 11th and 25th.*—Meeting *Chicago Association of Physicians and Surgeons*, in the evening, at Grand Pacific Hotel.

*Every Tuesday. Lectures.*—At Rush College, 9 to 1 o'clock.—Drs. Owens, Bridge, Adolphus and Parkes. At 4 P. M.—Dr. I. N. Danforth. At Chicago College, 8 to 12 o'clock.—Drs. Bond, Earle, Hutchinson and F. H. Davis. At Woman's Hospital College, 8 to 10 A. M.—Drs. McDonald and Bartlett.

*Clinics.*—At County Hospital, 2 P. M. Surgical.—Dr. Bogue. Medical. —Dr. Bevan. At Mercy Hospital, 3 P. M. Gynæcological.—Dr. Roler.

*Tuesday, 19th inst.—Illinois State Medical Society.* (Place announced by daily prints.)

*Tuesday, 12th inst.*—7.30 P. M., at 263 Wabash avenue, *Chicago Academy of Sciences.*

*Every Wednesday. Lectures.* At Rush College, 9 to 1 o'clock.—Drs. Wadsworth, E. F. Ingals, Strong and Parkes. At Chicago College, 8 to 12 o'clock. —Drs. Stilliaus, Sherman, Nelson and Merriman. At Woman's Hospital College, 9 to 11 A. M.—Drs. Thompson and Fisher.

*Clinics.*—At County Hospital, 2 P. M. Gynæcological.—Dr. Quine. 3 P. M. Ophthalmological.—Dr. Hotz. At Mercy Hospital, 3 P. M. Ophthalmological.—Dr. S. J. Jones. At St. Luke's Hospital, 8 A. M. Surgical.—Dr. Owens. At Central Dispensary, 3 P. M.—Dr. Bridge.

*Every Thursday. Lectures.*—At Rush College, 9 to 1 o'clock.—Drs. Hyde, Bridge, Jackson and Case. At Chicago College, 8 to 12 o'clock.—Drs. Quine, Earle, Hutchinson and Haines. At Woman's Hospital College, 3 to 6 P. M. Drs. Paoli, Blake and Delafontaine.

*Clinics.*—At Mercy Hospital, 3 P. M. Medical.—Dr. Merriman. At Central Dispensary, 2 P. M. Gynæcological.—Dr. Adolphus. Diseases of Chest. Dr. E. F. Ingals. At Hospital for Women and Children, 1.30 P. M. Medical. —Dr. A. H. Foster.

*Every Friday. Lectures.* At Rush College, 9 to 1 o'clock.—Drs. Wadsworth, Hayes, Adolphus and Case. At 4 P. M.—Dr. Danforth.

*Clinics.*—At County Hospital, 2 P. M. Medical.—Dr. Bevan. Surgical. —Dr. Bogue. At Mercy Hospital, 3 P. M. Medical.—Dr. Nelson.

*Every Saturday. Lectures.*—At Rush College, 9 to 12 o'clock.—Drs Owens, Hay and Strong. At Chicago College, 8 to 12 o'clock.—Drs. Bond, Jewell and Haines. At Woman's Hospital College, 10 to 12 o'clock.—Drs. Curtis and McDonald.

*Clinics.*—At Rush College, 2 P. M. Surgery.—Dr. Gunn. 3 P. M. Diseases of Nervous System.—Dr. Hay.

